# A family cluster of brucellosis in an underserved urban community in Guayaquil city, Ecuador

**DOI:** 10.3389/fpubh.2026.1863867

**Published:** 2026-08-18

**Authors:** Naomi Mora Jaramillo, Solon Alberto Orlando, Katherin Lucin, Fabiola Jimenez-Valenzuela, Andres Esteban Barragan-Peña, Angel Sebastian Rodriguez-Pazmino, Miguel Angel Garcia-Bereguiain

**Affiliations:** 1 Insituto Nacional de Salud Pública e Investigación, Guayaquil, Ecuador; 2 Universidad Ecotec, Samborondón, Ecuador; 3 Ministerio de Salud Pública, Guayaquil, Ecuador; 4 Universidad Católica Santiago de Guayaquil, Guayaquil, Ecuador; 5 One Health Research Group, Universidad de las Américas, Quito, Ecuador

**Keywords:** *Brucella abortus*, brucellosis, Ecuador, One Health, unpasteurized milk

## Abstract

Brucellosis is a neglected bacterial zoonosis caused by *Brucella* spp. with worldwide distribution. Infection typically occurs through direct contact with infected animals or consumption of unpasteurized dairy products, and the global burden of brucellosis is estimated over two million cases per year. In Latin America, brucellosis remains endemic, with important public health and economic consequences. Here, we report a family cluster of brucellosis in Guayaquil, Ecuador, involving six confirmed cases, including pediatric patients. We describe the clinical and epidemiological features of this family cluster and present *omp28* sequencing and phylogenetic analysis of the index case, highlighting the public health significance of *B. abortus* transmission in an underserved urban community. The epidemiological link of this urban family cluster to unpasteurized dairy products reinforced the need of an integrated One Health surveillance and control program from brucellosis beyond rural areas.

## Introduction

Brucellosis is a neglected bacterial zoonosis caused by *Brucella* spp., one of the most widespread diseases transmitted from animals to humans (1). Infection typically occurs through direct contact with infected animals, consumption of unpasteurized dairy products, or inhalation of contaminated aerosols (1, 2). The global burden of brucellosis is estimated at an annual incidence of over two million cases, with Africa and Asia carrying the highest burden, though the disease remains a persistent concern in Latin America (3–5). Human disease is most frequently attributed to *B. melitensis, B. abortus, B. suis*, and *B. canis*, each associated with distinct animal reservoirs and epidemiological patterns (4, 5).

In Latin America, brucellosis remains endemic, with important public health and economic consequences (6, 7). In Ecuador, bovine brucellosis alone generates annual agricultural losses estimated at over 5 million USD (8). According to national surveillance data from the Ministry of Public Health (MSP), a total of 133 human brucellosis cases have been reported nationwide over the last 5 years. Annual notifications show substantial variability, with 5 cases in 2020, 19 in 2021, 12 in 2022, 21 in 2023, 27 in 2024, and a marked rise to 49 cases in 2025. Throughout this period, cases were reported across multiple regions of the country, with Guayas, Imbabura, Pichincha and Manabí emerging as the most frequently affected provinces, with most cases affecting adults aged 20–49 years (9).

Human infections are often linked to rural exposures and consumption of artisanal dairy products, while urban outbreaks remain rare (10, 11). Clinically, brucellosis presents diagnostic challenges due to nonspecific symptoms such as undulant fever, fatigue, and musculoskeletal pain, which may lead to misdiagnosis and treatment delays (12, 13). Pediatric cases are particularly uncommon outside rural endemic regions, making their occurrence in urban settings of special epidemiological concern (14, 15).

Molecular epidemiology has become a cornerstone for outbreak investigation, enabling precise species identification, genetic characterization, and source attribution (16, 17). Among available molecular targets, the *omp28* gene (also known as *BP26*) is widely used for *Brucella* typing due to its species-level discriminatory power and relevance in diagnostic and vaccine research (18). However, data on the genetic diversity of *Brucella* in Ecuador remain limited, particularly in urban contexts. Here, we report a family cluster of brucellosis in Guayaquil, Ecuador, involving six confirmed cases, including pediatric cases. We describe the clinical and epidemiological features of the outbreak and present *omp28* sequencing and phylogenetic analysis of the index case, highlighting the public health significance of *B. abortus* transmission in an underserved urban community.

## Materials and methods

### Study setting and study population

The investigation was conducted in the parish of Pascuales, Guayaquil canton, Guayas province, Ecuador. Pascuales is an underserved urban area characterized by socioeconomic vulnerability, limited access to sanitation services and informal food distribution practices, which may facilitate zoonotic transmission pathways. The cases originated from this locality, and all epidemiological procedures were framed within this contextual setting. All the individuals included in the testing belonged to the same family, distributed in several households within the same neighborhood. After detection of the index case, an active surveillance protocol to seek for more cases was activated by public health authorities. Consumption of non-pasteurized dairy products was identified as the source of infection. A total number of 27 individuals belonging to the same family cluster were included in this surveillance based on confirmation of consumption of dairy products from the same provider, and 13 of those individuals presented symptoms compatible with brucellosis like fever, headache, diarrhea, abdominal pain, fatigue or weight loss. Those 13 symptomatic individuals were tested for *Brucella* infection, and also for *Leptospira* and arbovirus (Dengue and Chikungunya viruses, circulating arboviruses in the area at the time of infection).

### Sample collection

Blood samples were collected from the 13 individuals presenting clinical symptoms of brucellosis. Whole blood (1 mL) was obtained by venipuncture using EDTA tubes to prevent coagulation. Samples were transported under cold-chain conditions (2–8 °C) to “Instituto Nacional de Salud Pública e Investigación” (INSPI) in Guayaquil (Ecuador), where they were processed immediately upon arrival.

### *Brucella* spp. detection by PCR

Molecular detection of *Brucella* spp. was performed using conventional endpoint PCR targeting genus-specific markers; primers were selected according to Probert et al. (2003), specifically, the ARQ-070 primer pair was used (Forward: 5’-GCTCGGTTGCCAATATC AATGC-3′; Reverse: 5’-GGGTAAAGCGTCGCCAGAAG-3′) to amplify a genus-specific 151-bp fragment. For species identification, the GB1 (*omp28*) primer pair (Forward: 5’-GCCCCTGACATAACCC GCTT-3′; Reverse: 5’-GAGCGTGACATTTGCCGATA-3′) was employed to amplify a 1,030-bp fragment.

PCR reactions were performed in 25 μL total volume using GoTaq® Green Master Mix 2× (Promega, USA), which contains Taq DNA polymerase, dNTPs, MgCl₂, reaction buffer, and tracking dyes. The reaction mixture consisted of 12.5 μL of Master Mix, 0.5 μL of each primer (10 μM), 5 μL of template DNA, and nuclease-free water to final volume.

PCR amplification was conducted under the following thermal cycling conditions: initial denaturation at 95 °C for 5 min; 35 cycles of denaturation at 95 °C for 30 s, annealing at 57 °C for 1 min, and extension at 72 °C for 1 min; followed by a final extension at 72 °C for 5 min. Amplicons were visualized by electrophoresis on 2% agarose gels prepared in 1 × TAE buffer. Positive and negative controls were included in each PCR run to ensure assay validity.

### Sanger sequencing and phylogenetic analysis

PCR products corresponding to the *omp28* gene were purified using Exonuclease I and FastAP Thermosensitive Alkaline Phosphatase (Thermo Fisher Scientific, USA) to remove residual primers and dNTPs. Sequencing reactions were performed using the BigDye™ Terminator v3.1 Cycle Sequencing Kit (Applied Biosystems, USA), followed by purification via gel filtration with Sephadex™ G-100 (Cytiva, USA). Capillary electrophoresis was conducted on an ABI 3500 Genetic Analyzer (Applied Biosystems, USA) at the Sanger Sequencing Service, Universidad de las Américas, Quito, Ecuador.

Raw sequencing reads were processed using Geneious Prime software (Biomatters Ltd., New Zealand) for quality trimming, removal of low-quality regions, and clipping of secondary peaks. Forward and reverse sequences were aligned using ClustalW and assembled into consensus contigs. The resulting *omp28* sequence was compared against the GenBank database using BLAST to confirm species identification.

Phylogenetic analysis was performed using reference *omp28* sequences retrieved from GenBank, representing diverse *Brucella* species and geographically distinct *B. abortus* isolates. Multiple sequence alignment was conducted using ClustalW, and phylogenetic trees were constructed using the Maximum Likelihood (ML) method with the Kimura 2-parameter + Frequencies (K2P + F) evolutionary model. The robustness of tree topology was assessed through bootstrap analysis with 1,000 replicates. Phylogenetic reconstruction was performed using IQ-TREE software (version 2.0 or later), and tree visualization was conducted using FigTree (version 1.4.4).

### Statistical analysis

Positivity rates were calculated as the proportion of positive cases confirmed by PCR among the 27 exposed individuals. Confidence intervals (95%) for proportions were calculated using the exact binomial method.

## Results

### Brucellosis family cluster characterization

In February 2025, a case of human brucellosis was confirmed in Pascuales parish, Guayaquil, Ecuador, that was followed by several other cases within the same family cluster in households from the same neighborhood. Epidemiological research identified consumption of unpasteurized artisanal cheese purchased locally within the family cluster and originally brought from the rural canton of Vinces in the neighbor province of Los Rios. The affected family belonged to an underserved urban community characterized by socioeconomic vulnerability, limited sanitation infrastructure, and reliance on informal food distribution systems.

**Figure 1:**
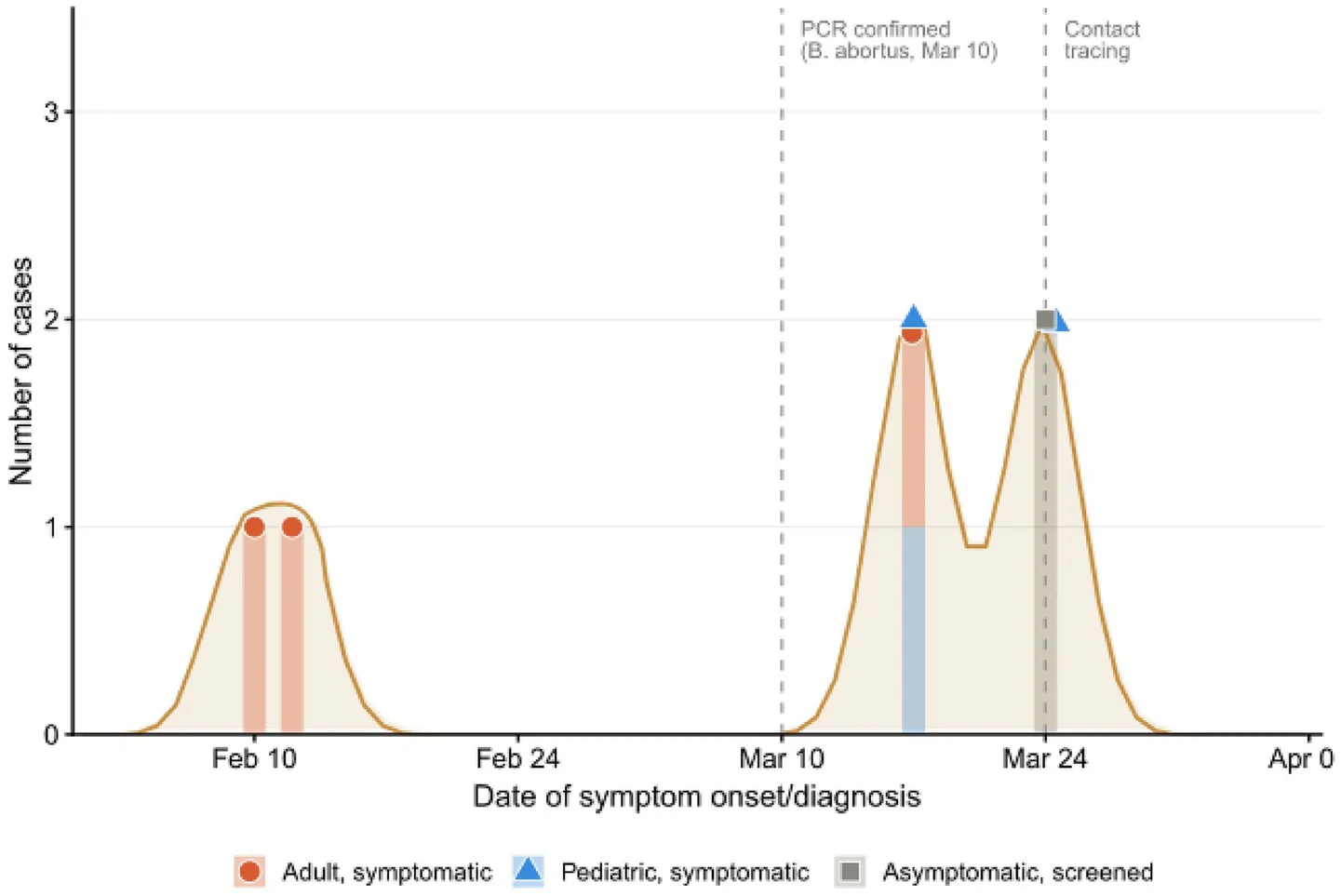
Epidemiological curve of the brucellosis family cluster described in this study.

The active surveillance was activated by public health authorities, and 27 member of the family were identified as consumers of unpasteurized artisanal cheese from the same origin. Of those, 27 individuals, 13 presented with symptoms of brucellosis in the following weeks (Figure 1, Table 1). Six of these individuals were confirmed as brucellosis cases by PCR, corresponding to a positivity rate for the total 27 exposed individuals of 22.2% (95% CI: 8.6–42.3%).

**Table 1:** Epidemiological information for the six positive cases un human brucellosis included in this study.

Age/Sex	Relationship	Onset	Key symptoms	Sampled	PCR+	Detection route
85M	Index case	Feb 10	Fever, chest pain, jaundice, hepatomegaly, dyspnea, weight loss	Feb 27	Mar 10	Hospitalized (ICU); discharged Mar 8
52F	Adult contact	Feb 12	Fever, diarrhea	Mar 24	Mar 25	Active contact tracing
60F	Adult contact	Mar 17	Fever, abdominal pain	Mar 24	Mar 25	Active contact tracing
10M	Pediatric contact	Mar 17	Fever, headache, and asthenia,	Mar 24	Mar 25	Active contact tracing
3M	Pediatric contact	Mar 24	Fever, malaise	Mar 24	Mar 25	Active contact tracing
3F	Pediatric contact	—	*Asymptomatic*	Mar 24	Mar 25	Active contact tracing

The chronological reconstruction of cases is detailed in Figure 1 and Table 1, and revealed that symptom onset among affected individuals occurred between February and March 2025, consistent with a point-source exposure. As stated above, the consumption of unpasteurized artisanal cheese distributed by a family member and purchased originally from the Vinces canton was identified as the most likely source of infection. However, direct microbiological testing of the implicated food product was not performed.

### Index case

The index case was an 85-year-old male residing in Pascuales parish, Guayaquil. He had medical history of hypertension, type 2 diabetes mellitus, and cardiomyopathy with left ventricular ejection fraction of 25%. He reported consuming unpasteurized artisanal cheese.

Clinical symptoms began on February 10, 2025, with a one-week clinical course characterized by precordial chest pain (pain scale 6/10), right hypochondrium pain, jaundice, anorexia, and weight loss of approximately 9–10 kg. Additional symptoms included dyspnea on moderate exertion, fever, cough, sore throat, diarrhea, headache, and night sweats.

On February 21, 2025, the patient was hospitalized at the internal medicine service in a hospital from Guayaquil city. Physical examination at admission revealed stable hemodynamics, jaundiced skin, dry oral mucous membranes, hypoventilation at lung bases with bilateral apical and left mid-lung dry velcro-type crackles, rhythmic hypophonetic heart sounds with a grade III mesocardiac murmur, active bowel sounds with hepatomegaly 1 cm below the costal margin and tenderness in the right hypochondrium, and lower extremity edema. The patient required intensive care unit admission. Blood samples were collected for laboratory testing of *Brucella*, *Leptospira*, dengue virus and chikungunya virus by PCR. The patient was only positive for *Brucella*.

### Pediatric cases

During household screening of exposed family members of the index case, three pediatric patients tested positive for brucellosis by PCR: a 10-year-old male, a 3-year-old male, and a 3-year-old female. The 10-year-old presented with fever, headache, and asthenia, while the 3-year-old male exhibited similar constitutional symptoms including fever and abdominal pain. The 3-year-old female remained asymptomatic throughout the investigation period. None of the pediatric cases developed severe complications or required hospitalization. Moreover, blood samples were also collected for laboratory testing of *Leptospira*, dengue virus and chikungunya virus by PCR in these patients, yielding negative results for these 3 pathogens.

All three pediatric cases were epidemiologically linked to the index case through household contact and shared exposure to unpasteurized artisanal cheese consumed within the family. The children received age-appropriate antibiotic regimens and demonstrated complete clinical recovery without relapse during follow-up evaluation.

### Molecular detection and species identification

Out of 13 blood samples collected from the symptomatic individuals, six tested positive for *Brucella* DNA by PCR, confirming a brucellosis family cluster. Among these, only the index case yielded sufficient DNA quality for extended molecular characterization with *omp28* gene. Amplification and sequencing of the *omp28* gene generated a 932 bp sequence that was identified as *Brucella abortus* by BLAST alignment against reference sequences. Regarding the failure to obtain good quality DNA sequences from five of six positive cases, this was probably affected by two factors: (1) household contacts were sampled 7–14 days post-onset, when *Brucella* bacteremia is known to decline; (2) samples were received in the National Institute of Health after 48 h since collection and break of cold chain cannot totally be excluded.

**Figure 2:**
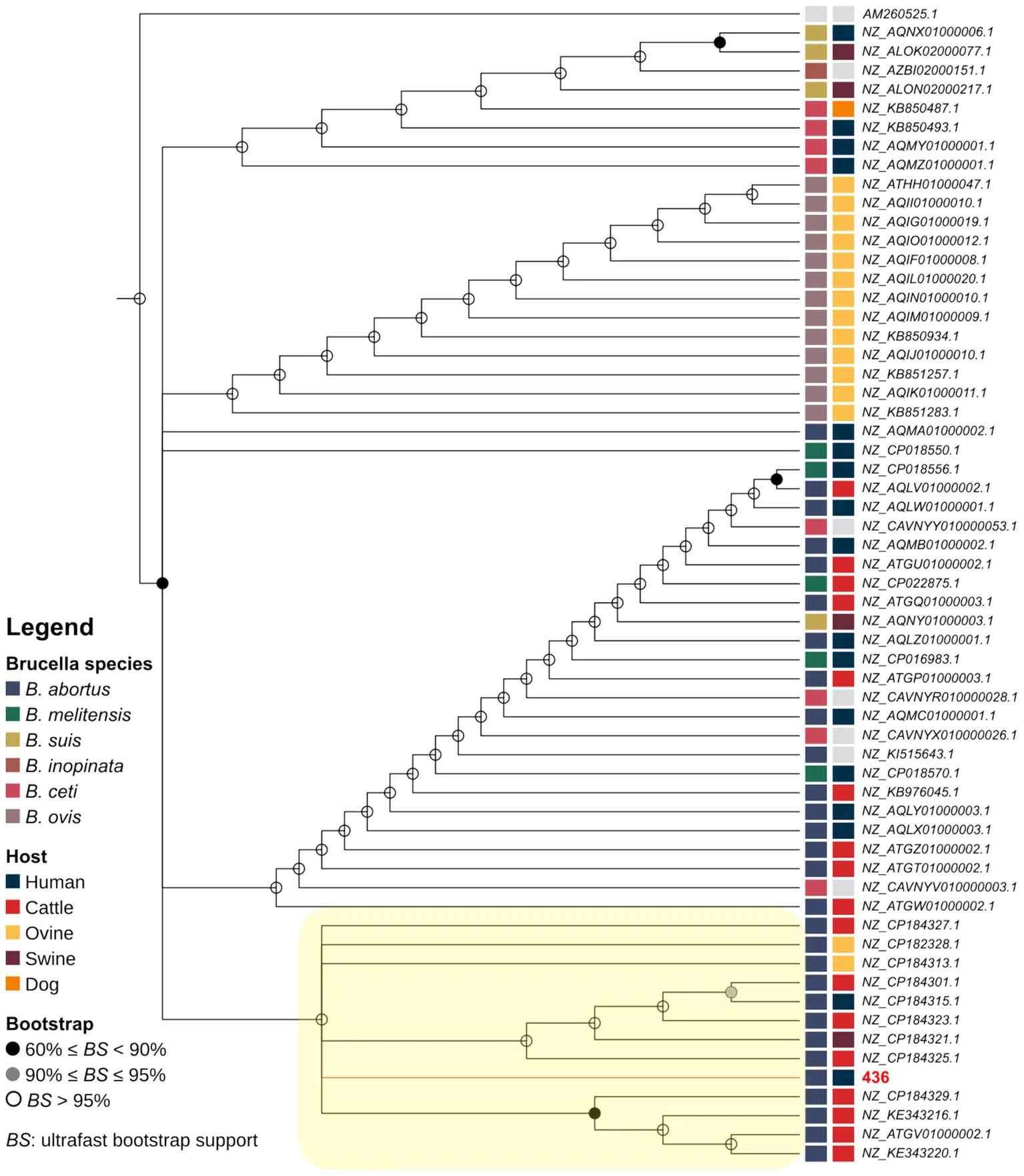
Maximum likelihood phylogenetic tree of *Brucella* spp. based on the *omp28* gene (932 bp). The isolate from the index case in this study is shown in red (highlighted within yellow box). *Brucella* species and host origin of each strain is shown by colored boxes.

The *omp28* sequence from the index case clustered firmly within the *B. abortus* clade, highlighted in Figure 2 (yellow box). Within this clade, the isolate from Ecuador grouped closely with reference strains of *B. abortus* isolated mainly from cattle and one sequence from humans in other geographic regions. The clustering was supported by high bootstrap values (>95%), confirming the robustness of the placement. No evidence of divergence toward other *Brucella* species (*B. melitensis* or *B. suis*) was observed.

## Discussion

The current study represents the first molecularly confirmed family cluster of brucellosis in an underserved urban setting in Ecuador. The identification of *Brucella* spp. by conventional PCR but also *Brucella abortus* identification by *omp28* gene sequencing in the sample from the index case underscores the critical value of molecular epidemiology for zoonotic outbreak investigation in resource-limited contexts. Molecular tools enable precise species identification and genetic characterization, thereby supporting targeted public health interventions (18, 19).

Comparable applications of molecular methods have been reported in China, Turkey, and India, where sequencing of *omp28, omp31*, and other diagnostic markers was used to confirm human infections and trace transmission within family or community clusters (18–21). Similarly, in Latin America, molecular typing has proven crucial for differentiating *Brucella* species in animal reservoirs and tracking cross-species transmission (6, 22, 23). In Ecuador, prior molecular studies have focused mainly on livestock isolates, identifying *B. abortus* as the predominant species and revealing molecular patterns consistent with long-term endemic circulation in bovine herds, reporting, for the first time in Ecuador, the detection of biovar 2 in bovine herds (22). However, our investigation is the first study implementing these techniques in humans, which emphasizes the urgency of strengthening diagnostic capabilities for timely detection of outbreaks in vulnerable groups.

The molecular characterization of the index case isolate through *omp28* gene sequencing provided valuable insights into the genetic relationship and epidemiological context of this outbreak. The sequence obtained from the index case showed definitive clustering within the *B. abortus* clade with high bootstrap support (>95%), confirming the robustness of species identification. This clustering with bovine and human isolates from other regions suggests a conserved genetic lineage consistent with bovine-to-human transmission (24, 25). The epidemiological evidence, in this case the consumption of unpasteurized cheese from a bovine brucellosis endemic region in Ecuador, would suggest bovine-human transmission route, although the lack of microbiological confirmation of Brucella in that cheese is an important limitation. Such foodborne exposure remains the primary mechanism for human brucellosis worldwide (26–28). Although the omp28/BP26 locus is a validated target for *Brucella* species identification with high specificity and sensitivity in human blood um samples, this conserved gene cannot resolve strain clustering between human cases. An important limitation of this study was that neither MLVA-16 nor WGS could be performed for strain and clustering characterization, and implementing this methods would be a fundamental priority for future outbreak investigations in Ecuador.

Brucellosis remains endemic in Ecuador, with underreported human cases due to diagnostic and surveillance limitations (29, 30). The Ministry of Public Health estimated an incidence of 0.16 per 1,00,000 inhabitants in 2024, though the true burden is likely higher (31). Importantly, the persistence of human infection is closely linked to the wide circulation of *Brucella* among multiple animal reservoirs in the country. Studies in cattle report seroprevalence values ranging from 6.2 to 23.8% depending on production system, geographic region and vaccination coverage (10, 22). Free-roaming dogs and wild mammals have also been identified as potential reservoirs of *Brucella* in Ecuador (32–34). These findings underscore that brucellosis is endemic in Ecuador and present in diverse animal hosts, facilitating spillover into vulnerable human populations. Although we approached regional veterinary authorities to request information about brucellosis prevalence in the area of production of the suspected contaminated cheese, that information was not available.

Our findings highlight how socioeconomic vulnerability and informal food production networks can sustain zoonotic transmission even in urban settings. Similar urban outbreaks have been documented in other low- and middle-income countries where sanitary deficiencies and a lack of surveillance persists (26, 34–36). The clinical presentation among affected family members, predominantly nonspecific febrile illness, reflects the diagnostic challenges of brucellosis (4, 12). The severe course observed in the older adult index case contrasts with the mild or asymptomatic infections in pediatric contacts, consistent with previous observations of variable disease expression across age groups (13, 37).

Our findings expose structural deficiencies in brucellosis control programs. Despite existing national regulations (38), Ecuador lacks comprehensive data on bovine vaccination coverage and traceability of dairy products. The persistence of artisanal cheese markets without sanitary oversight continues to pose a major public health risk. Strengthening veterinary vaccination programs and establishing traceable, pasteurized dairy supply chains should be priorities to prevent future outbreaks (10, 39). Brucellosis, like all zoonoses, requires a holistic, multidisciplinary, integral approach under the One Health scheme, which recognizes the interdependence between human, animal, and environmental health (40). Various studies highlight that the presence of outbreaks in vulnerable communities is closely related to sanitary deficiencies in livestock herds and the lack of integrated epidemiological surveillance (32, 34). In this sense, public health interventions in the community to prevent the consumption of raw milk or derived dairy products are strongly recommended, alongside with improving molecular testing for routine diagnosis at laboratories in Ecuador.

This study have several limitations, some of them already acknwledge but overall summarized here: (1) Important information related to antibiotic treatment and case management is missing, especially for the interesting pediatric cases; (2) The absence of microbiological testing for *Brucella* in the suspected cheese did not allow to fully support bovine-human transmission; (3) The inability to sequence additional cases due to low DNA yield and the absence of higher-resolution genotyping methods like MLVA or WGS did not allowed strains and clusters characterization to sequencing to better elucidate transmission pathways and genetic diversity (20, 41, 42); (4) The lack of epidemiological information in the region of study about the burden of brucellosis in livestock is a handicap to promote a One Health approach to prevent further outbreaks.

In conclusion, this urban family cluster of *B. abortus* infection in Guayaquil highlights critical surveillance gaps and reaffirms the value of molecular epidemiology in zoonotic outbreak detection. Strengthening diagnostic infrastructure, enforcing dairy safety measures, and adopting a One Health approach are essential for mitigating the reemergence of brucellosis in Ecuador and other endemic regions.

## Data Availability

All relevant data is included within the manuscript. Any additional data request will be provided upon request to the authors.
